# Live attenuated VZV vaccination induces antitumor immunity in ATLL patients

**DOI:** 10.1007/s00262-022-03301-6

**Published:** 2022-10-01

**Authors:** Tatsuro Jo, Ritsuko Kubota-Koketsu, Yohei Kaneko, Takahiro Sakai, Kazuhiro Noguchi, Sadaharu Irie, Masatoshi Matsuo, Jun Taguchi, Kuniko Abe, Kazuto Shigematsu

**Affiliations:** 1 Department of Hematology, Japanese Red Cross Nagasaki Genbaku Hospital, Nagasaki, Japan; 2 Department of Viral Infections, Research Institute for Microbial Diseases, Osaka University, Osaka, Japan; 3 Department of Laboratory, Japanese Red Cross Nagasaki Genbaku Hospital, Nagasaki, Japan; 4 Department of Pharmacy, Japanese Red Cross Nagasaki Genbaku Hospital, Nagasaki, Japan; 5 Department of Pathology, Japanese Red Cross Nagasaki Genbaku Hospital, Nagasaki, Japan

**Keywords:** Adult T cell leukemia, Lymphoma, ATLL, Varicella-zoster virus vaccine, VZV, HTLV-1 Tax, T cell receptor repertoire

## Abstract

Adult T cell leukemia/lymphoma (ATLL) is a CD4-positive peripheral T cell lymphoma caused by human T cell lymphotropic virus type 1 (HTLV-1). Although ATLL is quite difficult to be cured, up-regulation of cellular immunity such as HTLV-1 Tax-specific cytotoxic T lymphocytes (CTLs) has been proved to be important to obtain long-term survival. At present, no efficacious method to activate ATLL-specific cellular immunity is available. This study aimed to investigate whether live attenuated varicella-zoster virus (VZV) vaccination to ATLL can activate HTLV-1 Tax-specific cellular immune response. A total of 3 indolent- and 3 aggressive-type ATLL patients were enrolled. All aggressive-type patients had the VZV vaccination after completing anti-ATLL treatment including mogamulizumab, which is a monoclonal antibody for C–C chemokine receptor 4 antigen, plus combination chemotherapy, whereas all indolent-type patients had the VZV vaccination without any antitumor treatment. Cellular immune responses including Tax-specific CTLs were analyzed at several time points of pre- and post-VZV vaccination. After the VZV vaccination, a moderate increase in 1 of 3 indolent-type patients and obvious increase in all 3 aggressive-type patients in Tax-specific CTLs percentage were observed. The increase in the cell-mediated immunity against VZV was observed in all indolent- and aggressive-type patients after VZV vaccination. To conclude, VZV vaccination to aggressive-type ATLL patients after mogamulizumab plus chemotherapy led to the up-regulation of HTLV-1 Tax-specific CTLs without any adverse event. Suppression of regulatory T lymphocytes by mogamulizumab may have contributed to increase tumor immunity in aggressive-type ATLL patients. Japan Registry of Clinical Trials number, jRCTs051180107.

## Introduction

The retrovirus human T cell lymphotropic virus type 1 (HTLV-1) infects CD4-positive T lymphocytes, and the HTLV-1 genome encodes the transcriptional activator proteins Tax and HBZ that are critical for immortalization and genetic instability of HTLV-1-infected T lymphocytes [1–3]. After HTLV-1 infection gradually obtains various genetic abnormalities, adult T cell leukemia/lymphoma (ATLL) develops in many decades [4]. ATLL is clinically subdivided into 4 types: smoldering, chronic, lymphoma, and acute [5]. Despite intensive chemotherapies, the prognosis of aggressive-type ATLL subtypes (acute, lymphoma, and poor risk chronic) remains quite poor. A randomized phase 3 trial that compared VCAP-AMP-VECP therapy (VCAP, vincristine, cyclophosphamide, doxorubicin, and prednisolone; AMP, doxorubicin, ranimustine, and prednisolone; and VECP, vindesine, etoposide, carboplatin, and prednisolone) with biweekly CHOP therapy (cyclophosphamide, doxorubicin, vincristine, and prednisolone) revealed that overall 3-year survival rates were 24% and 13%, respectively [6]. Although mogamulizumab (MOGA), a monoclonal antibody for C–C chemokine receptor 4 (CCR4) antigen, that acts via antibody-dependent cellular cytotoxicity was allowed to use for aggressive-type ATLL in combination with chemotherapies such as VCAP-AMP-VECP and EPOCH (etoposide, vincristine, doxorubicin, cyclophosphamide, and prednisolone), no survival benefit was obtained to date [7, 8]. After obtaining a complete response (CR) with first-line chemotherapy, allogeneic hematopoietic stem cell transplantation (allo-HSCT) can be considered as an effective treatment option [9]. Tax can be a target for the host immune system especially cytotoxic T lymphocytes (CTLs). Actually, human leukocyte antigen (HLA)-A2-, HLA-A24-, and HLA-A11-restricted Tax-specific CTLs (TaxCTLs) were reported in some ATLL patients who were treated by allo-HSCT [10, 11]. Furthermore, TaxCTLs were often observed in patients with MOGA-induced skin disorders; a statistically significant overall survival benefit was observed in patients with MOGA-induced skin disorders compared with those without skin disorders [8]. Therefore, induction of TaxCTLs may lead to survival benefit for ATLL patients. We recently reported some ATLL cases with maintenance of memory TaxCTLs; they had herpes virus infection during the chemotherapy period or at just completing chemotherapy with or without MOGA and obtained long-term survival [12]. On the other hand, although HTLV-1 encodes the *gag*, *pol,* and *env* gene regions, the products of these genes such as internal proteins, reverse transcriptase, and outer coat proteins as well as HBZ coded in the *pX* region, are not the main targets of CTLs [13]. Currently, a live attenuated VZV vaccine (OKA/Biken live attenuated varicella VZV vaccine; BIKEN, Osaka, Japan) is available for prevention of varicella and is used worldwide [14, 15]. This vaccine (vaccine OKA) is a live attenuated virus obtained by culturing varicella-zoster virus (parental OKA) isolated from a child with typical varicella at 34 °C for 11 passages in human embryonic lung cells, followed by 12 passages in guinea pig embryonic fibroblast cells, and then by passaging in human diploid cells [16]. The varicella vaccine is effective not only in preventing varicella but also herpes zoster, and activation of T cell-mediated cellular acquired immunity has been shown to play an important role [17–20]. Furthermore, an attempt to test the use of viral products as interferon inducers in various carcinomas as anticancer immunotherapy was reported. Of the 25 subjects tested, two (one with adult acute leukemia and another one with pediatric acute lymphoblastic leukemia) were reported to have achieved therapeutic outcomes [21]. As mentioned above, TaxCTLs have been reported in some ATLL patients who obtained long-term survival after HSCT or (immuno)chemotherapy [8–12]. Therefore, we hypothesized that varicella-zoster virus (VZV) vaccination could induce TaxCTLs in ATLL patients. To analyze the disease state of ATLL in chronological order, TCR *V beta (Vb)* gene repertoire, soluble interleukin 2 receptor (sIL-2R), abnormal lymphocytes, and HTLV-1 provirus load were used along with TaxCTLs as evaluation items.

## Materials and methods

### Patients

The inclusion criteria for the study are as follows: (1) HTLV-1 antibody is positive. The diagnosis is made as a peripheral lymphoid tumor cytologically or histopathologically. (2) CCR4 antigen of the tumor is positive in flow cytometric analysis or immunohistochemical analysis. Laboratory tests to confirm CCR4 antigen expression were performed using the POTELIGEO Test FCM kit (cat. 58691-3, MINARIS MEDICAL, Japan) for flow cytometry and the POTELIGEO Test IHC kit (cat. 58687-6, MINARIS MEDICAL, Japan) for immunohistochemical staining, according to the manufacturer’s instructions. (3) An anti-VZV antibody [immune adherence hemagglutination (IAHA) or enzyme immunoassay (EIA)] is positive (more than 4 times) before VZV vaccine inoculation, to prevent extremely rare serious adverse reactions such as disseminated varicella, antibody-negative subjects were excluded for safety reasons in this study. (4) It is confirmed that cellular immunocompetence of patients is enough [with positive results of lymphocyte stimulation test for phytohemagglutinin (PHA) and concanavalin-A (Con-A)]. (5) The patients have HLA type A*02:01 or A*24:02. (6) Permitted if already started on MOGA monotherapy or MOGA plus chemotherapy. (7) The patients at the age of 20 years or older can be enrolled. (8) Ineligible for hematopoietic stem cell transplantation, or the patient does not wish to receive it.

The exclusion criteria for the study are as follows: (1) In the case that the patient develops a herpes virus infectious disease after the initiation of the immunochemotherapy. (2) In the case that the patient has an active overlapping cancer. (3) In the case that the patient is positive for hepatitis virus type B antigen. (4) In the case that the patient is positive for human immunodeficiency virus antibody (5) Pregnant and lactating women. (6) In the case that the patient has a psychosis or psychiatric condition that makes it difficult to participate in the study. (7) In the case that the patient has another disease resulting in immunodeficiency or immunosuppressive status and is receiving an immunosuppressive therapy. (8) In the case that the principal or subprincipal investigators consider the patient unsuitable for study enrollment due to serious complications other than those listed above.

Notably, 3–5 patients each with indolent- and aggressive-type ATLL were planned to be enrolled for 3.5 years from May 1, 2016. As a result, 3 indolent- and 3 aggressive-type ATLL patients were enrolled. Written informed consent was obtained from all participants.

The study was approved by Japanese Red Cross Nagasaki Genbaku Hospital Ethical Review Board (approval number: 406), the BIKEN institutional Ethical Review Board (approval number: 16-01), and Shiga University of Medical Science Clinical Research Review Board (approval number: L2018-008) and was conducted in accordance with the Declaration of Helsinki (registered number: UMIN000021750 and jRCTs051180107).

### Treatment

All 3 aggressive-type ATLL patients were treated with MOGA plus chemotherapy, whereas indolent-type ATLL patients were not treated with any agents. CHOP, VCAP-AMP-VECP, and EPOCH therapies were used in this study as the chemotherapy regimens [6, 8, 22]. The CHOP regimen consists of doxorubicin (50 mg/m^2^ at day 1), cyclophosphamide (750 mg/m^2^ at day 1), vincristine (1.4 mg/m^2^, maximum 2 mg at day 1), and prednisolone (40 mg/m^2^ at days 1–5). The VCAP-AMP-VECP (mLSG15) regimen consists of the following 3 regimens: VCAP (vincristine, 1 mg/m^2^, maximum 2 mg; cyclophosphamide, 350 mg/m^2^; doxorubicin, 40 mg/m^2^; and prednisolone, 40 mg/m^2^) at day 1; AMP (doxorubicin, 30 mg/m^2^; ranimustine, 60 mg/m^2^; and prednisolone, 40 mg/m^2^) at day 8; and VECP (vindesine, 2.4 mg/m^2^ at day 15; etoposide, 100 mg/m^2^ at days 15–17; carboplatin, 250 mg/m^2^ at day 15; and prednisolone, 40 mg/m^2^ at days 15–17). The EPOCH regimen consists of continuous intravenous infusions at days 1–4 of etoposide (50 mg/m^2^), vincristine (0.4 mg/m^2^), and doxorubicin (10 mg/m^2^), with bolus doses of cyclophosphamide (750 mg/m^2^ at day 6) and oral prednisolone (60 mg/m^2^ at days 1–6).

Aggressive-1 patient was treated with 6 cycles of MOGA plus CHOP. Aggressive-2 patient was treated with 3 cycles of MOGA plus mLSG15 and then 2 cycles of MOGA plus EPOCH. The Aggressive-3 patient was treated with 3 cycles of MOGA plus mLSG15, 1 cycle of MOGA plus EPOCH, and then 1 cycle of MOGA monotherapy. The regimen alteration from mLSG15 to EPOCH was made owing to bone marrow suppression by mLSG15 in Aggressive-2 and Aggressive-3 patients. CR was confirmed before VZV vaccination in all aggressive-type patients.

### Humoral and cellular immunity at the study entry

Before VZV vaccination, anti-VZV antibody titration by EIA and IAHA for humoral immunity and lymphocyte blastoid transformation (LBT) test by PHA and Con-A stimulation for cellular immunity were conducted in all patients. All of these laboratory tests were performed by SRL corporation (Tokyo, Japan).

### VZV vaccination

**Fig. 1:**
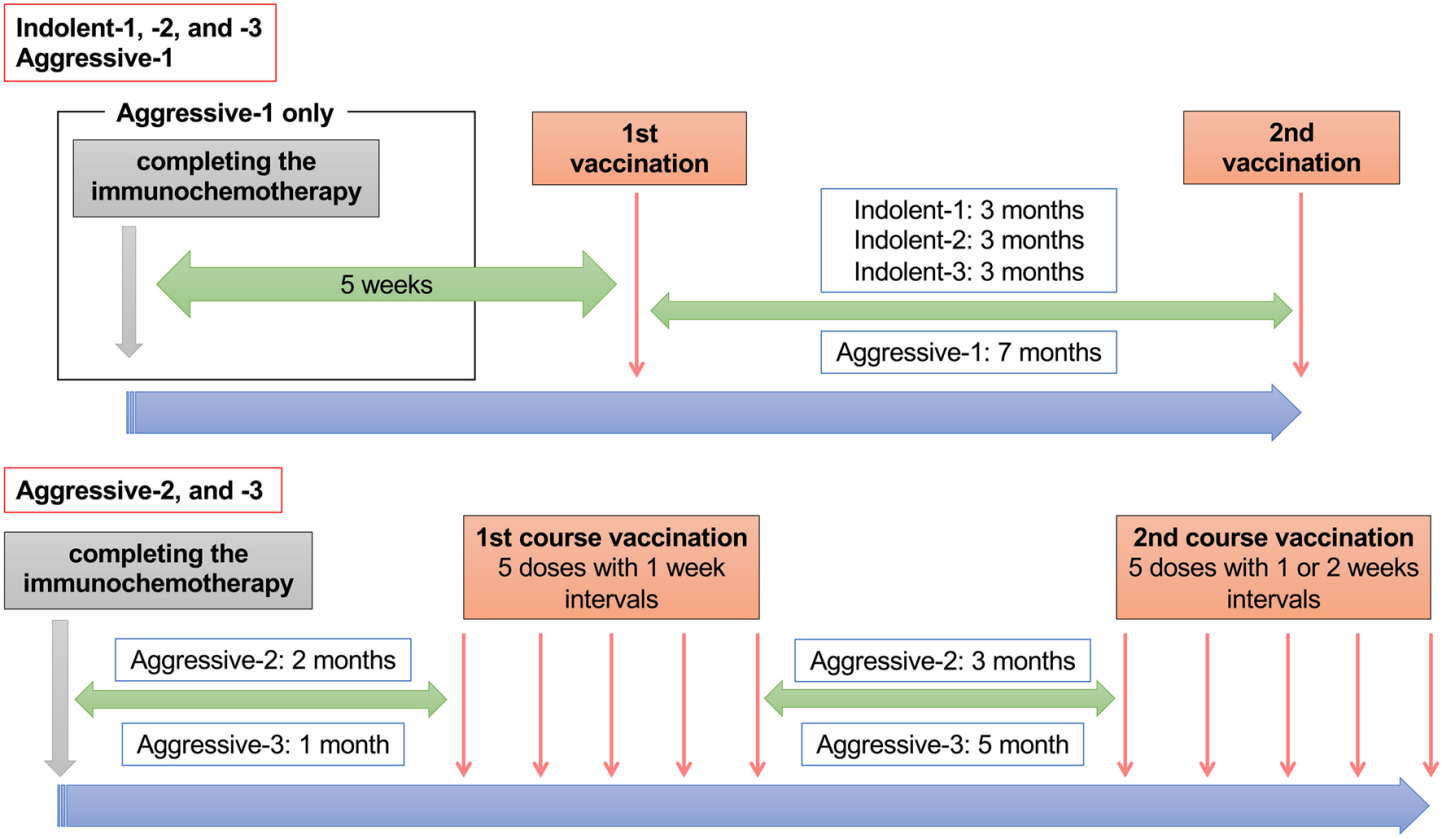
Live attenuated VZV vaccination schedule. Live attenuated VZV vaccine (OKA/Biken live attenuated varicella VZV vaccine; BIKEN, Osaka, Japan) was inoculated twice with at least a 12-week interval between 2 doses. The vaccination method was changed in patients enrolled after May 2017 to 2 courses; each course included up to 5 doses of vaccine with 1–4-weeksinterval. This alteration was applied to Aggressive-2 and Aggressive-3 patients

Live attenuated VZV vaccine (OKA/Biken live attenuated varicella VZV vaccine; BIKEN, Osaka, Japan), developed in 1974 [14, 15], is a lyophilized preparation of the OKA/Biken strain of live attenuated VZV. Each 0.5 mL dose contains a minimum of 1000 plaque-forming units of VZV when reconstituted [23, 24]. The vaccine was inoculated twice with at least 12 weeks interval between 2 doses. The VZV vaccination of four patients (Indolent-1, -2, -3, and Aggressive-1) did not cause any adverse reactions. Therefore, the VZV vaccination method was changed for patients enrolled after May 2017 to 2 courses to intensify immunization; each course included up to 5 doses of vaccine with 1–4-weeks intervals. The aggressive-type patients had VZV vaccination after the end of the whole courses of immunochemotherapy, whereas the indolent-type patients had VZV vaccination without any antitumor treatment (Fig. 1).

### Analysis of biomarkers

Each patient’s HLA was determined using peripheral blood mononuclear cells (PBMNCs) by SRL corporation (Tokyo, Japan). PBMNCs were drawn from each patient and HLA-A*02:01-restricted and HLA-A*24:02-restricted TaxCTLs were analyzed without any stimulation. HLA-A*02:01-restricted Tax tetramer (HTLV-1 Tax 178–186 Peptide, MBL, Nagoya, Japan) and HLA-A*24:02-restricted Tax tetramer (HTLV-1 Tax 301–309 Peptide, MBL, Nagoya, Japan) were used for detecting Tax-specific CTLs. The percentages of CD4- and CD8-positive T lymphocytes in PBMNCs and percentages of abnormal lymphocytes in white blood cells were measured. The sIL-2R and HTLV-1 provirus load were measured by SRL corporation (Tokyo, Japan). The Beta Mark TCR Vb repertoire kit (Beckman Coulter, Tokyo, Japan) was used to analyze TCR *Vb* gene repertoire of total CD8-positive T lymphocytes by flow cytometry according to the manufacturer’s instructions. These analyses were conducted at several time points of pre-VZV vaccination and 2 weeks, 45–60 days, 3 months, 6 months, and 12 months after completing the VZV vaccination.

### Skin test and VZV-specific cell-mediated immunity (CMI)

Skin test for VZV antigen and whole-blood stimulation with VZV antigen (ex vivo) to measure VZV-specific CMI were conducted. Varicella virus antigen (BIKEN, Osaka, Japan) was used for skin test. Briefly, 0.1 mL of varicella virus antigen was injected intradermally into the forearm. The erythematous change was judged 48 h after injection. The longest diameter was used for the test results. Ex vivo VZV-specific CMI was performed at BIKEN according to a previously described method [25]. Briefly, heparinized blood corrected from the patients were stimulated by incubation with a live VZV at 37 °C and 5% CO_2_ for 9 days. For responder measurement, the cells were stained with the following antibody cocktail: anti-CD3-BV785 (cat. 3017330, BioLegend, USA), anti-CD4-PC7 (cat. 6607101, Beckman Coulter, USA), annti-CD45RA-BV421 (cat. 304129, BioLegend, USA), anti-CD45RO-PE (cat. A07787, Beckman Coulter, USA), and anti-CD197 (CCR7)-APC (cat. 353214, BioLegend, USA), and measured on the flow cytometer. VZV-specific CD4^+^ memory T cells were defined as the CD3^+^CD4^high^CD45RA^−^RO^+^ lymphocytes. VZV-specific CD8^+^ effector T cells were defined as the CD3^+^CD4^−^CD45RA^+^RO^+^ lymphocytes, and VZV-specific CD8^+^ memory T cells were defined as the CD3^+^CD4^−^CD45RA^−^RO^+^ lymphocytes.

## Results

### Patient characteristics

The patients’ characteristics are presented in Table 1. The positivity of anti-VZV antibody was confirmed by EIA and IAHA assays in all patients. The IAHA assay was not conducted in Aggressive-3. In all patients, sufficient cellular immunity was also confirmed by LBT test.

**Table 1:** Patient characteristics at the point of study entry

Patient	Indolent-1	Indolent-2	Indolent-3	Aggressive-1	Aggressive-2	Aggressive-3
ATLL subtype	Smoldering	Smoldering	Smoldering	Acute	Lymphoma	Acute
Age	59	67	66	71	68	60
Sex	Male	Male	Male	Male	Female	Male
HLA	A*02:06:01 A*24:02	A*24:02	A*24:02	A*11:01 A*24:02	A*01:01:01 A*02:01:01	A*24:02
*Anti-VZV antibody*						
EIA	47.7	14.3	49.3	45.1	10.8	12.8
IAHA	32	16	64	64	16	Not done
*LBT (cpm)*						
PHA	19,900	27,000	23,700	6530	17,900	13,800
Con-A	18,600	18,300	19,600	5780	19,900	12,100
Control	308	753	1570	203	107	368

Standard value of LBT by PHA: 20,500–56,800 cpm. Standard value of LBT by Con-A: 20,300–65,700 cpm. Standard value of LBT control: 127–456 cpm

EIA, Enzyme immunoasaay (negative: < 2.0); IAHA, immune adherence hemagglutination (negative: < 2 ×); LBT, lymphocyte blastoid transformation; cpm: counts per minute; PHA, phytohemagglutinin; Con-A, concanavalin-A

### Analysis of TaxCTLs

**Fig. 2:**
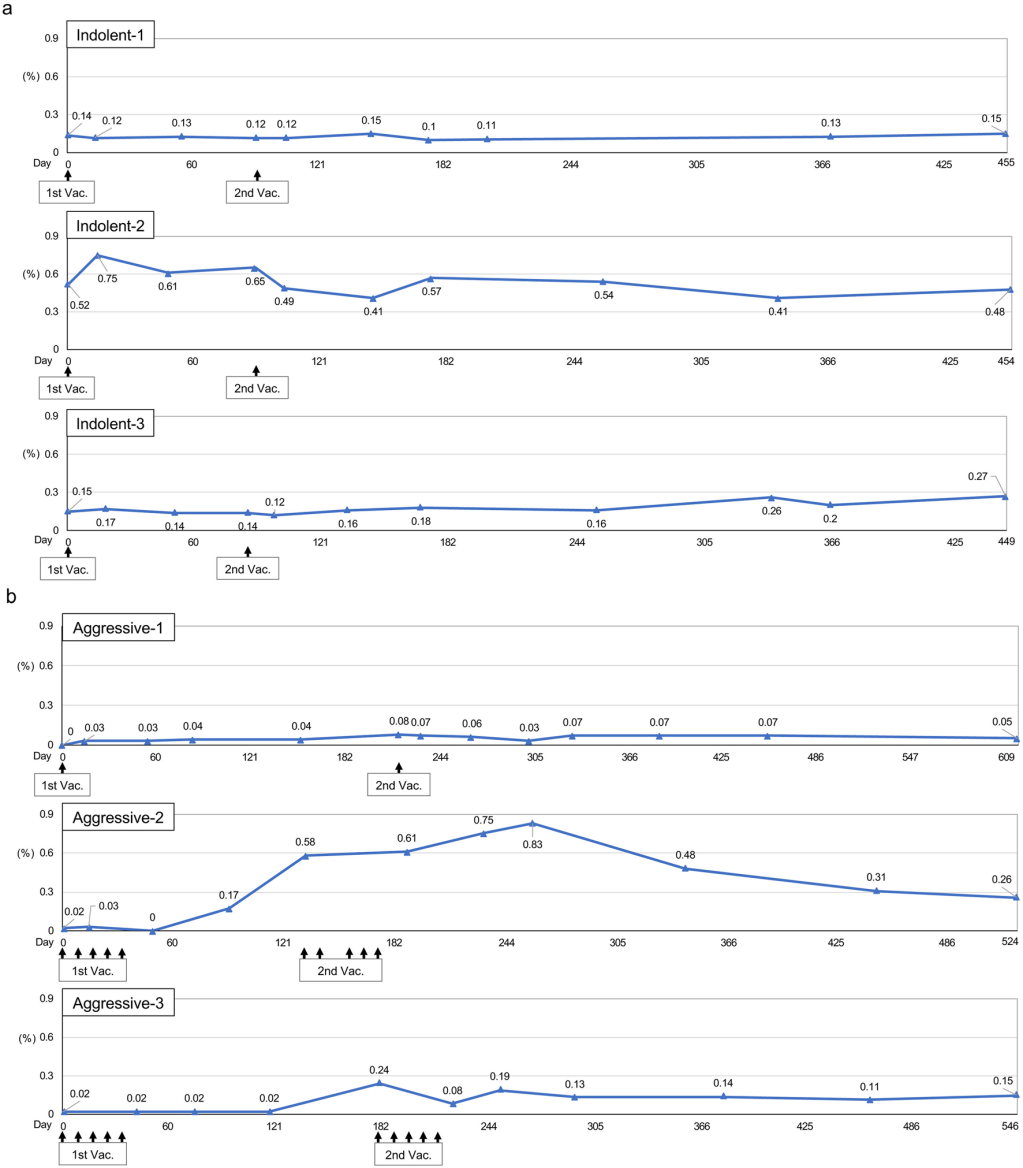
Changes in HTLV-1 Tax-specific CTLs. Percentages of Tax-specific CTLs in CD8-positive lymphocytes were indicated. **a** Indolent-type ATLL patients. The upper, middle, and lower panels indicate data of Indolent-1, Indolent-2, and Indolent-3, respectively. **b** Aggressive-type ATLL patients. The upper, middle, and lower panels indicate data of Aggressive-1, Aggressive-2, and Aggressive-3, respectively. 1st Vac., first VZV vaccination; 2nd Vac., second VZV vaccination

Changes in TaxCTLs are presented in Fig. 2. At the study entry, the CTLs were already recognized with relatively high levels in all indolent-type patients. There was no significant change in the CTLs observed by VZV vaccination in Indolent-1. Before the first VZV vaccination, a high percentage of the CTLs (0.52%) were observed in Indolen-2. The value elevated to 0.75% transiently after the first vaccination, but the value returned to the original level (0.48%) during the end of the study. In Indolent-3, the percentages of the CTLs gradually elevated after the second vaccination from 0.15% at the study entry to 0.27% at the end of the study (Fig. 2a).

Before the first VZV vaccination, the values of TaxCTLs were 0 or very low (0.02%) in all aggressive-type patients. After the vaccination, the CTLs were clearly induced and maintained until the end of the study in Aggressive-1. Significant elevation of the CTLs (0.17%) was observed after approximately 3 months after the first vaccination in Aggressive-2. The value of the CTLs continued to increase up to 0.83% even after second vaccination and then gradually decreased but were maintained at a high level (0.26%) at the end of the study. A similar pattern in Aggressive-2 was observed in Aggressive-3. The value of the CTLs definitely increased (0.24%) after approximately 6 months after the first vaccination. After second vaccination, the CTLs were maintained with high levels until the end of the study (Fig. 2b).

### Changes of other biomarkers

**Fig. 3:**
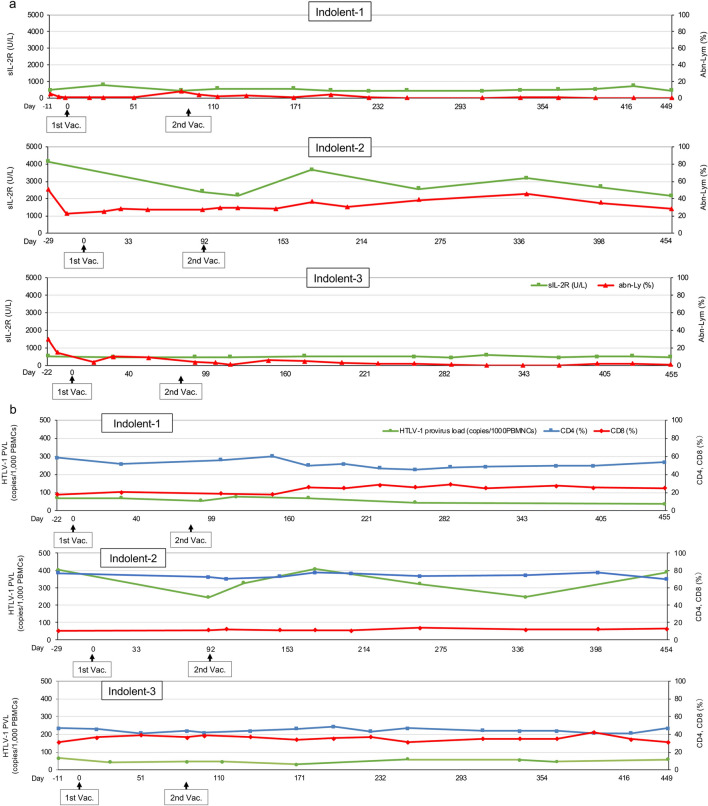

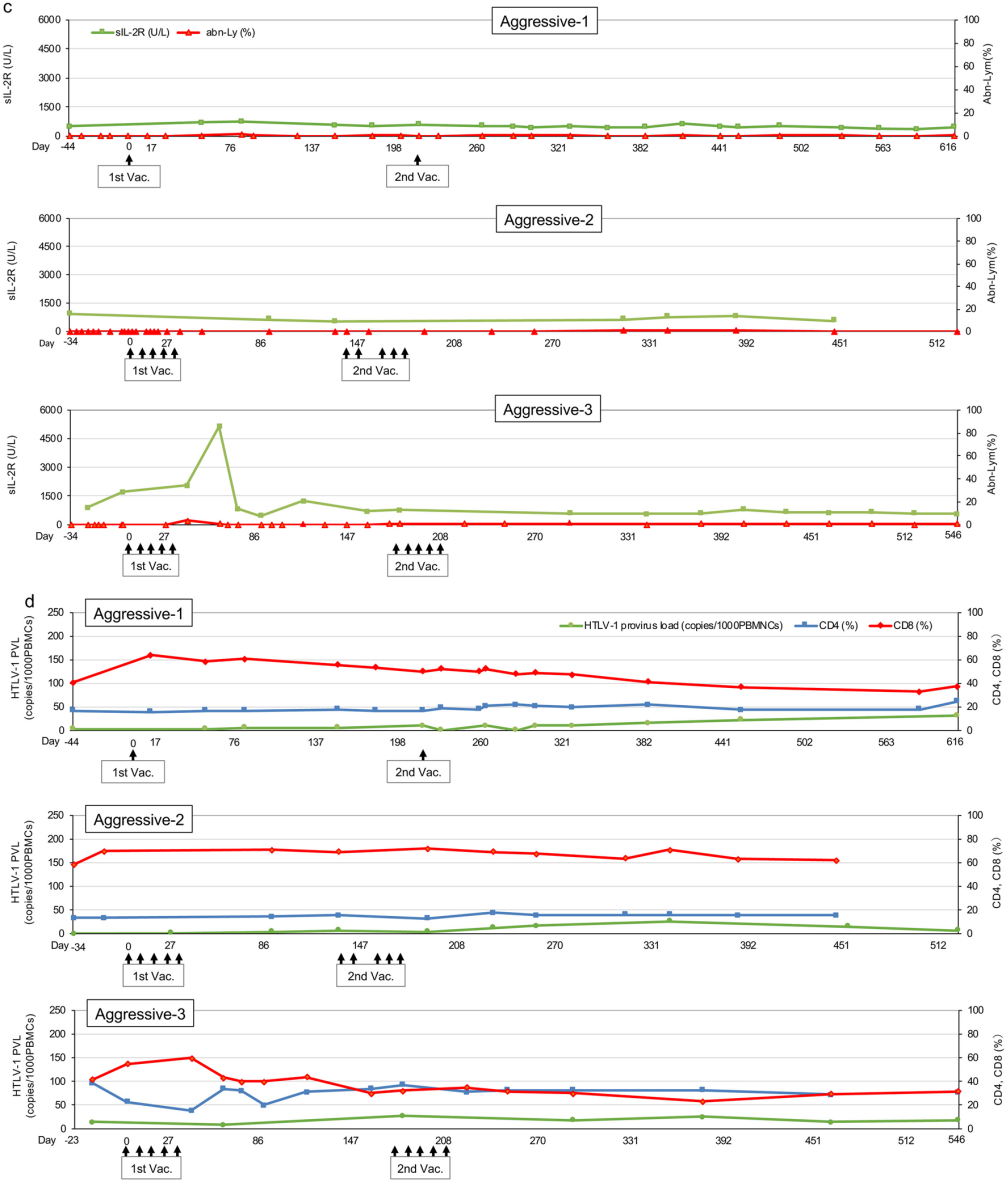
Changes in several biomarkers. **a** Values of sIL-2R and the percentages of abnormal lymphocytes in peripheral blood in indolent-type ATLL patients. Green lines: sIL-2R (U/L). Red lines: abnormal lymphocytes (abn-Ly) per white blood cells in peripheral blood (%). The upper, middle, and lower panels indicate data of Indolent-1, Indolent-2, and Indolent-3, respectively. **b** HTLV-1 provirus load (copies/1000PBMNCs) and the percentages of CD4- and CD8-positive T lymphocytes in peripheral blood in indolent-type ATLL patients. Green lines: HTLV-1 provirus load (copies/1000PBMNCs). Blue lines: CD4^+^ T cells per lymphocytes in peripheral blood (%). Red lines: CD8^+^ T cells per lymphocytes in peripheral blood (%). The upper, middle, and lower panels indicate data of Indolent-1, Indolent-2, and Indolent-3, respectively. **c** Values of sIL-2R and the percentages of abnormal lymphocytes in peripheral blood in aggressive-type ATLL patients. Green lines: sIL-2R (U/L). Red lines: abnormal lymphocytes (abn-Ly) per white blood cells in peripheral blood (%). The upper, middle, and lower panels indicate data of Aggressive-1, Aggressive-2, and Aggressive-3, respectively. **d** HTLV-1 provirus load (copies/1000PBMNCs) and the percentages of CD4- and CD8-positive T lymphocytes in aggressive-type ATLL patients. Green lines: HTLV-1 provirus load (copies/1000PBMNCs). Blue lines: CD4^+^ T cells per lymphocytes in peripheral blood (%). Red lines: CD8^+^ T cells per lymphocytes in peripheral blood (%). The upper, middle, and lower panels indicate data of Aggressive-1, Aggressive-2, and Aggressive-3, respectively. 1st Vac., first VZV vaccination; 2nd Vac., second VZV vaccination

After the VZV vaccination, the percentages of peripheral blood abnormal lymphocytes reduced to 0 or quite low values in Indolent-1 and Indolent-3, whereas the rate was not so changed in Indolent-2 (Fig. 3a). The values of sIL-2R were approximately 500 U/L at the study entry in Indolent-1 and Indolent-3, and the values were not changed until the end of the study, whereas the value in Indolent-2 was approximately 4000 U/L and reduced by half after the VZV vaccination (Fig. 3a). The values of HTLV-1 provirus load were not changed by the VZV vaccination in all indolent-type patients (Fig. 3b). Reduction of the gaps between CD4- and CD8-positive T lymphocyte percentages was observed in Indolent-1 and Indolent-3 (Fig. 3b).

Throughout the study, the percentages of peripheral blood abnormal lymphocytes were 0 or quite low values in all aggressive-type patients (Fig. 3c). The values of sIL-2R were maintained below 1000 U/L throughout the study in Aggressive-1 and Aggressive-2. In Aggressive-3, the values of sIL-2R transiently increased up to 5140 U/L after the first VZV vaccination owing to viral infection and inflammation of right external ear canal that were not considered adverse events of the VZV vaccination. After the second VZV vaccination, the values remained below 1000 U/L until the end of the study (Fig. 3c). Throughout the study, the values of HTLV-1 provirus load were below 40 copies/1000 PBMNCs in all aggressive-type patients (Fig. 3d). Reversal of the CD4-to-CD8 ratio was observed at the study entry in all aggressive-type patients, and the status was almost maintained throughout the study (Fig. 3d). The values of sIL-2R and percentages of CD4- and CD8-positive T lymphocytes were not measured at the 12-month time point from the second VZV vaccination by an accident in Aggressive-2.

### Analysis of effector and memory CTLs using TCR Vb gene repertoire assay

**Fig. 4:**
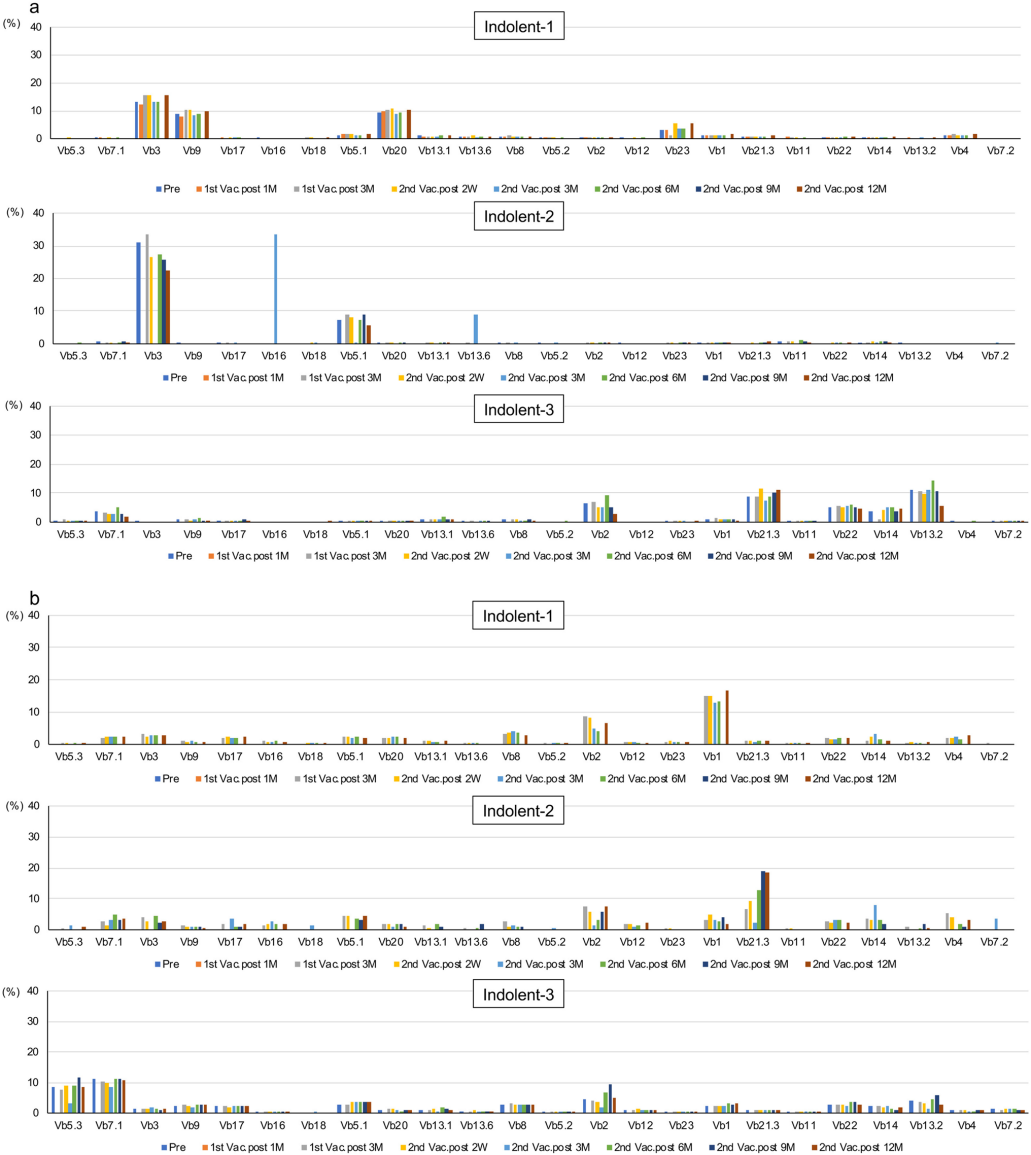

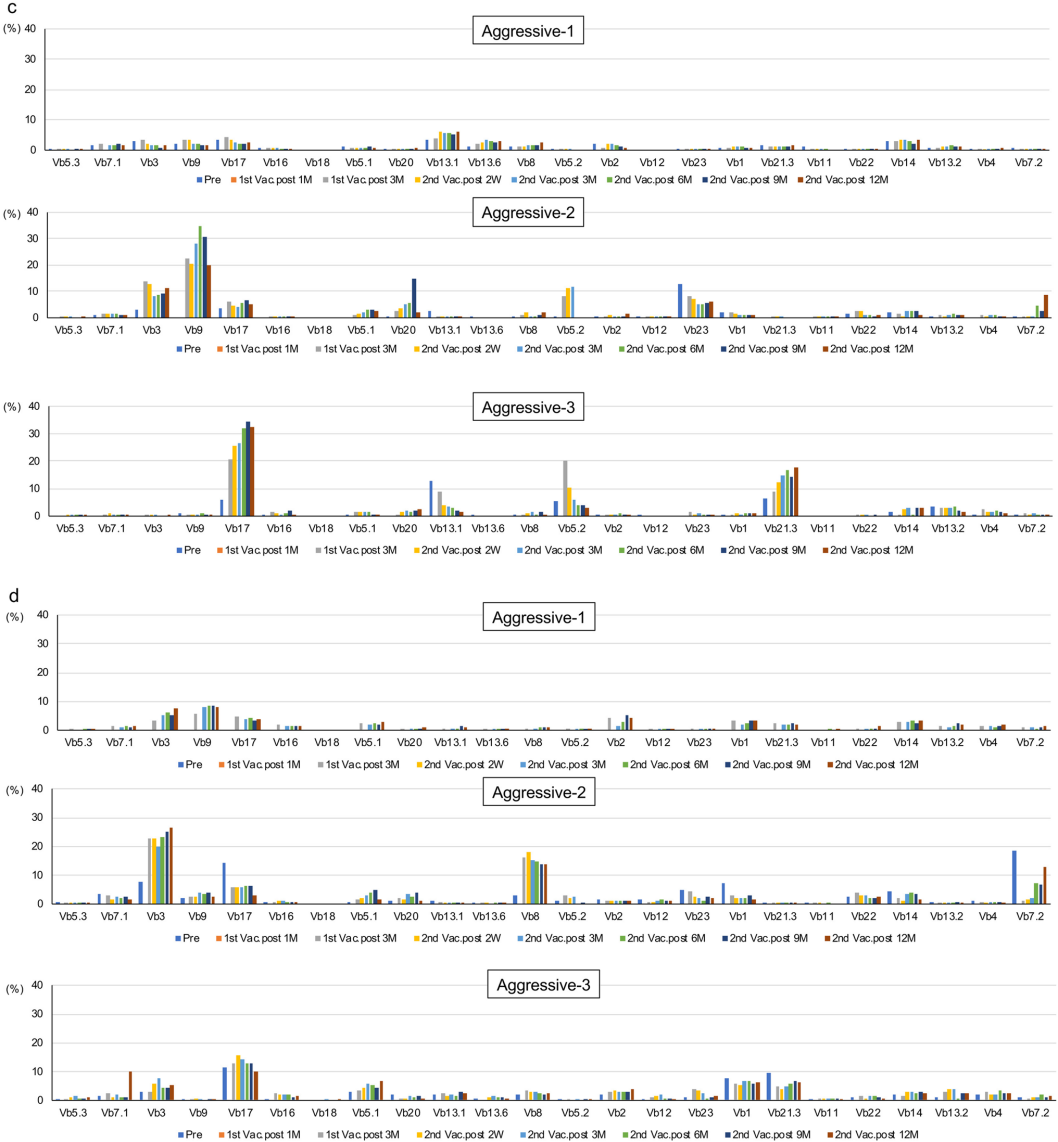
Analysis of the T cell receptor *V beta* gene repertoire. **a** Effector CTLs in indolent-type ATLL patients. The upper, middle, and lower panels indicate data of Indolent-1, Indolent-2, and Indolent-3, respectively. **b** Memory CTLs in indolent-type ATLL patients. The upper, middle, and lower panels indicate data of Indolent-1, Indolent-2, and Indolent-3, respectively. **c** Effector CTLs in aggressive-type ATLL patients. The upper, middle, and lower panels indicate data of Aggressive-1, Aggressive-2, and Aggressive-3, respectively. **d** Memory CTLs in aggressive-type ATLL patients. The upper, middle, and lower panels indicate data of Aggressive-1, Aggressive-2, and Aggressive-3, respectively. Vb, *V beta* gene; Pre, Pre-VZV vaccination; 1st Vac., First VZV vaccination; 2nd Vac., Second VZV vaccination; M, Month(s) after VZV vaccination; 2 W, 2 weeks after VZV vaccination

The results of TCR receptor Vb repertoire analysis are presented in Fig. 4. A few effector CTL clones already existed at the study entry in all indolent-type patients. These CTL clones were maintained after the VZV vaccination without no remarkable change until the end of the study (Fig. 4a). A few memory CTL clones, such as CTLs using TCR *Vb2* and *Vb1* in Indolent-1 and CTLs using TCR *Vb2* and *Vb21.3* in Indolent-2, were induced and maintained after the VZV vaccination (Fig. 4b). Two memory CTL clones using TCR *Vb5.3* and *Vb7.1* were observed before the first VZV vaccination in Indolent-3. These CTLs were maintained after the VZV vaccination until the end of the study (Fig. 4b).

In Aggressive-1, no significant effector CTL clone was observed throughout the study. However, a few memory CTL clones, such as CTLs using *Vb3* and *Vb9*, were induced and maintained after the VZV vaccination (Fig. 4c, d). In Aggressive-2, a few effector CTL clones, such as CTLs using TCR *Vb3* and *Vb9*, were induced and maintained after the VZV vaccination (Fig. 4c), whereas a few memory CTLs, such as CTLs using TCR *Vb3* and *Vb8*, were induced and maintained after the VZV vaccination (Fig. 4d). In Aggressive-3, 2 effector CTL clones using *Vb17* and *Vb21.3* were activated and maintained after the VZV vaccination (Fig. 4c), whereas a few memory CTL clones already existed before the first vaccination and they were maintained throughout the study (Fig. 4d).

### Analysis of skin test and ex vivo VZV-specific CMI

**Fig. 5:**
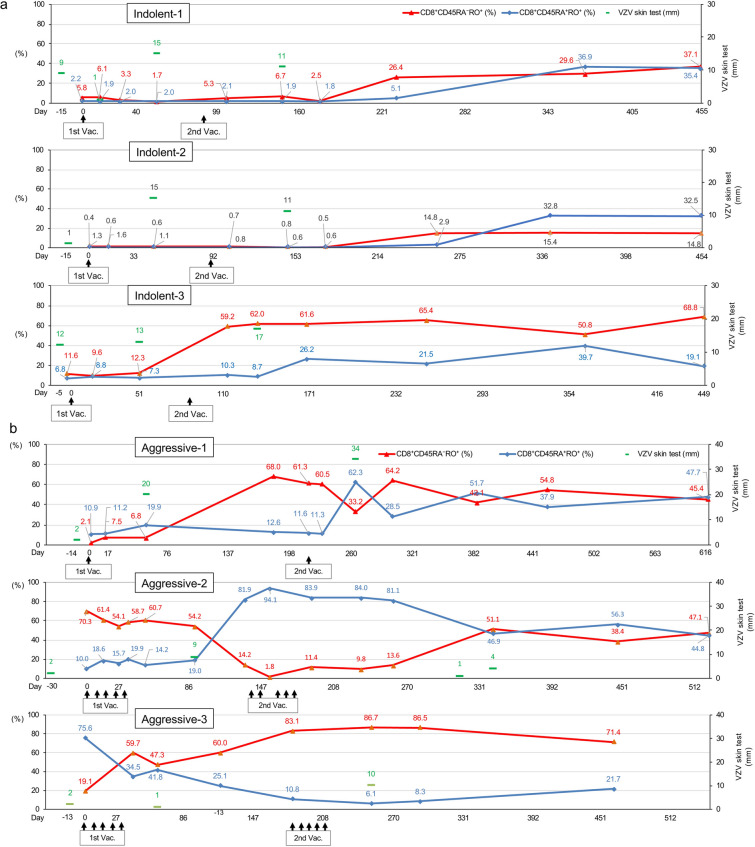

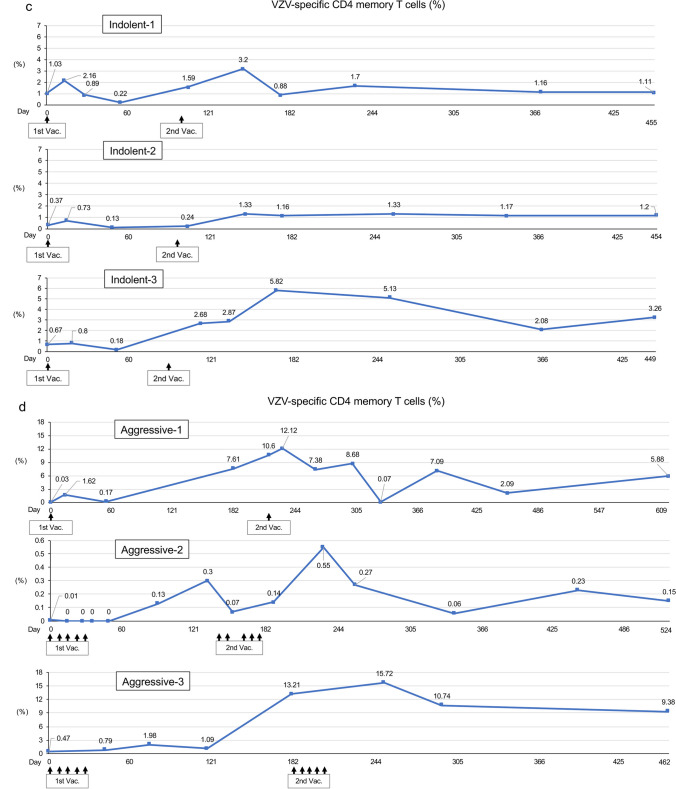
Changes in VZV skin test, VZV-specific CD8^+^ T cells, and VZV-specific CD4^+^ memory T cells. **a** VZV skin test and VZV-specific CD8^+^ T cells in the indolent-type ATLL patients. The upper, middle, and lower panels indicate data of Indolent-1, Indolent-2, and Indolent-3, respectively. Green bar: the major axis (millimeter) of skin redness by VZV skin test. Red lines: percentages of VZV-specific CD8^+^ memory T cells (CD3^+^CD4^−^CD45RA^−^RO^+^ population) in CD8-positive T lymphocytes. Blue lines: percentages of VZV-specific CD8^+^ effector T cells (CD3^+^CD4^−^CD45RA^+^RO^+^ population) in CD8-positive T lymphocytes. **b** VZV skin test and VZV-specific CD8^+^ T cells in the aggressive-type ATLL patients. The upper, middle, and lower panels indicate data of Aggressive-1, Aggressive-2, and Aggressive-3, respectively. Green bar: the major axis (millimeter) of skin redness by VZV skin test. Red lines: percentages of VZV-specific CD8^+^ memory T cells (CD3^+^CD4^−^CD45RA^−^RO^+^ population) in CD8-positiveT lymphocytes. Blue lines: percentages of VZV-specific CD8^+^ effector T cells (CD3^+^CD4^−^CD45RA^+^RO^+^ population) in CD8-positive T lymphocytes. **c** VZV-specific CD4^+^ memory T cells in indolent-type ATLL patients. The upper, middle, and lower panels indicate data of Indolent-1, Indolent-2, and Indolent-3, respectively. **d** VZV-specific CD4^+^ memory T cells in aggressive-type ATLL patients. The upper, middle, and lower panels indicate data of Aggressive-1, Aggressive-2, and Aggressive-3, respectively. 1st Vac., First VZV vaccination; 2nd Vac., Second VZV vaccination

Skin tests with VZV antigen were conducted for a few time points at pre- and post-VZV vaccination (Fig. 5a, b). The long axis of skin redness became larger after VZV vaccination in all indolent- and aggressive-type patients. Because the production of the VZV antigen for the skin test was stopped by the manufacturer, the skin tests must have been ceased in the middle of the study.

A significant elevation of VZV-specific CD4^+^ memory T cells were observed after the VZV vaccination in all patients (Fig. 5c, d).

The percentages of both effector and memory VZV-specific CD8^+^ T cells were low at the study entry, but elevation of those cells was observed after the VZV vaccination in all indolent-type patients (Fig. 5a). Data on the effector and memory VZV-specific CD8^+^ T cells in the aggressive-type patients are presented in Fig. 5b. Similar to the indolent-type patients, both effector and memory VZV-specific CD8^+^ T cells elevated after the VZV vaccination in Aggressive-1 patient. In Aggressive-2 patient, the VZV-specific CD8^+^ effector T cells showed a similar movement pattern of Tax-specific CTLs (Fig. 2b). The value of VZV-specific CD8^+^ effector T cells was low (10.0%) before the first VZV vaccination and elevated at a high level (94.1%) after the second vaccination. Then, the values decreased but were maintained with high levels (more than 44%) until the end of the study. However, the value of VZV-specific CD8^+^ memory T cells was high (70.3%) before the first vaccination. Contrary to the CD8^+^ effector T cells, the CD8^+^ memory T cells decreased to 1.8% after the first vaccination and gradually elevated and were maintained with high levels as much as the CD8^+^ effector T cells until the end of the study. In Aggressive-3 patients, VZV-specific CD8^+^ memory T cells rapidly elevated to a high level (59.7%) after the first VZV vaccination, and the value further elevated up to 86.7% after the second vaccination and the CD8^+^ T cells were maintained with high level (71.4%) at the final examination. Both VZV-specific CD4^+^ and CD8^+^ T cells were not measured at the 12-month time point after the second VZV vaccination because of the limitation of supported period form March 27, 2018, to December 31, 2019, by BIKEN (Osaka, Japan).

### Adverse events and the disease status at the end of the study

No intolerable adverse event was observed after the VZV vaccination. All indolent-type patients did not transform into aggressive-type ATLL until the end of the study, and all aggressive-type patients had maintained CR throughout the study.

## Discussion

Before MOGA was used to treat ATLL, we had experienced some aggressive-type ATLL patients who had herpes infection after obtaining CR and long-term survival [12]. TaxCTLs were maintained for a long period in the patients. However, HTLV-1 provirus was not eradicated even in that condition. These results meant that HTLV-1 was not eliminated even in long-term survivors with TaxCTLs. Moreover, these results suggest that herpes virus infection may up-regulate anti-ATLL immunity simultaneously with antiherpes virus immunity and that the up-regulated anti-ATLL immunity including TaxCTLs may exert to kill ATLL cells with chemotherapy agents and to inhibit relapse of ATLL for a long period. It is impossible to afflict ATLL patients with herpes virus infection after obtaining CR as desired. Therefore, we planned this study to examine whether live attenuated VZV vaccination, instead of herpes virus infection, can activate TaxCTLs in patients with ATLL.

The reasons why treatment naïve indolent-type patients were included in this study are as follows: (1) The prognosis of indolent-type patients was not necessarily good. The mean overall survival was only 2.9 years without plateau [26]. (2) No efficacious treatment regimen was reported to date [27]. (3) If VZV vaccination can activate TaxCTLs, it may result in the inhibition of transformation in disease status from indolent to aggressive form.

If aberrant gene products, which are essential for cancer cell survival, have strong immunogenicity, cancer cells with such genetic abnormalities should be excluded by the host's immune surveillance systems. Therefore, the immunogenicity of the abnormal gene products present in the resulting cancer may be very weak, and the frequency of such tumor-specific CTLs is expected to be very low [28]. As a result, it is generally difficult to directly identify such tumor antigen-specific CTLs ex vivo, and they can only be detected after expansion by repeated in vitro stimulation with the antigenic peptide on appropriate antigen-presenting cells. However, the data in this study on TaxCTLs were surprising. A moderate increase in TaxCTLs in 1 of 3 indolent-type patients and definite increases in all 3 aggressive-type patients were observed by the VZV vaccination without any adverse event (Fig. 2). Interestingly, TaxCTLs were observed with relatively high values before the VZV vaccination in all indolent-type patients, whereas the values of the CTLs were 0 or quite low in all aggressive-type patients. These results suggest that the decrease of TaxCTLs may be relevant to the progression in the disease status to acute form in indolent-type patients. Relatively high values of TaxCTLs at the study entry may be one of the reasons why they did not change the values of TaxCTLs by VZV vaccination in Indolent-1 and Indolent-2. However, TCR repertoire assays revealed that selected memory CTL clones, such as CTLs using *Vb1* in Indolent-1 and *Vb2* in Indolent-2, were activated after the VZV vaccination (Fig. 4b). A few effector and memory CTL clones were definitely activated after VZV vaccination and up-regulation of TaxCTLs in all aggressive-type patients (Figs. 2b, 4c, d). No exacerbation in the values of sIL-2R, abnormal lymphocytes, and HTLV-1 provirus load was observed after the VZV vaccination in all indolent- and aggressive-type patients (Fig. 3). These results suggest that VZV vaccination may activate cellular immunity including anti-ATLL immunity even in indolent- and aggressive-type patients.

Why TaxCTLs were more likely to be activated in aggressive-type patients than in indolent-type patients? There are a few possible reasons: (1) The disease status was controlled by immunochemotherapy including MOGA only in aggressive-type patients. Therefore, the aggressive-type patients remained to be in a more sensitive condition to VZV vaccination than in the indolent-type patients. (2) Reversal of the CD4-to-CD8 ratio had been already obtained with immunochemotherapy at the study entry only in all aggressive-type patients (Fig. 3d). The indolent-type patients were at a disadvantage in low rates of CD8 compared with the aggressive-type patients (Fig. 3b). (3) CCR4-positive regulatory T lymphocytes were thought to be reduced at the study entry in all aggressive-type patients by MOGA plus chemotherapy. This suggests that aggressive-type patients were likely to be under conditions that made it easier to activate cellular immunity compared with indolent-type patients. Furthermore, this speculation suggests that MOGA followed by VZV vaccination procedure may be an effective treatment option for indolent-type ATLL patients.

An attempt using Tax peptide-pulsed dendritic cell vaccine for previously treated ATLL patients has been recently reported [29]. This therapy expects treatment effects by TaxCTLs in an HLA-restricted manner. This approach demands a relatively long and complicated process; as a result, the vaccine therapy can be conducted in limited facilities. In contrast, our VZV vaccination method after MOGA plus chemotherapy in aggressive-type ATLL patients is a simple procedure and HLA unrestricted, which means all ATLL patients can be candidates for the VZV vaccination method. Furthermore, our VZV vaccination method can be conducted in almost all general hospitals.

Even if TaxCTLs are activated and amplified by VZV vaccination, an important issue is whether these CTLs have sufficient killing ability against ATLL tumor cells. It has been reported that Tax is substantially expressed at sites where ATLL cells are actively proliferating, but in quiescent conditions, such as in PB, Tax expression is extremely low and cannot be identified by flow cytometry, but can only be detected by reverse transcription polymerase chain reaction (RT-PCR) [30]. However, Tax-specific CTLs have been shown to recognize HLA/Tax peptide complexes on the cell surface of ATLL cells and have cytotoxic activity against a variety of HTLV-1-infected cells even when Tax expression is extremely low that could be only detectable by RT-PCR [30, 31]. Therefore, it is quite possible that TaxCTLs activated and amplified by VZV vaccination may play a role in the treatment and prevention of recurrence of ATLL. In fact, the disease conditions did not worsen in Indolent-1 and all three aggressive-type patients during the course of this study. On the other hand, it has been reported that the level of Tax expression in HTLV-1-infected cells decreases during disease progression, and Tax transcripts are detected only in approximately 40% of established ATLL cases [13, 32]. However, during the decades following HTLV-1 infection until the onset of ATLL, a substantial amount of genetic and epigenetic abnormalities accumulates in HTLV-1-infected cells [3, 13, 33]. Some of these abnormalities may be recognized as tumor antigens by host immunocompetent cells, as is the case with Tax. It has been reported that interferon gamma production is increased during herpesvirus infection or VZV vaccination, resulting in activation of cellular immunity [34, 35]. Therefore, even in ATLL patients who do not express any Tax, cellular immunity activated by VZV vaccination may react to tumor antigens other than Tax and produce antitumor effects. Furthermore, it is theoretically possible that vaccines other than the live attenuated VZV vaccine we used in this study could produce therapeutic responses similar to those observed in this study, as long as the vaccine is capable of sufficiently activating cellular immunity.

More than 90% of adults have been infected by VZV and are at risk of herpes zoster by the reactivation of latent VZV [36]. It was suggested that VZV-specific cellular immunity may play an important role in preventing herpes zoster [37, 38]. Although the live attenuated VZV vaccine can be used in adults at the age of more than 50 years to prevent herpes zoster, it is not recommended for use in immunocompromised persons [39]. In this study, the live attenuated VZV vaccination was safely administered and up-regulated VZV-specific effector and memory CTLs in adult patients with ATLL (Fig. 5). These results suggest that the live attenuated VZV vaccination can be safely administered in adult patients with malignancy under some conditions, such as with the positivity of anti-VZV antibodies and proof of sufficient cellular immunity by LBT tests.

## Conclusion

The live attenuated VZV vaccination after MOGA plus chemotherapy was safely conducted and led to definite up-regulation of TaxCTLs in all aggressive-type ATLL patients. This immune intensification may play a role in combination with immunochemotherapy and may exert to inhibit relapse of ATLL. Although only 1 of 3 indolent-type patients exhibited a moderate elevation of TaxCTLs, a few memory CTL clones were recognized by TCR repertoire assays in other 2 indolent-type patients. The VZV vaccination after MOGA treatment, leading to a decreased number of regulatory T lymphocytes, may become a possible treatment option for indolent-type ATLL via up-regulation of TaxCTLs.

## Data Availability

The datasets generated and analyzed during the current study are available from the corresponding author on reasonable request.
